# PCSK9 in Myocardial Infarction and Cardioprotection: Importance of Lipid Metabolism and Inflammation

**DOI:** 10.3389/fphys.2020.602497

**Published:** 2020-11-12

**Authors:** Ioanna Andreadou, Maria Tsoumani, Gemma Vilahur, Ignatios Ikonomidis, Lina Badimon, Zoltán V. Varga, Péter Ferdinandy, Rainer Schulz

**Affiliations:** ^1^Laboratory of Pharmacology, Faculty of Pharmacy, National and Kapodistrian University of Athens, Athens, Greece; ^2^Cardiovascular Program-ICCC, Research Institute-Hospital de la Santa Creu i Sant Pau, IIB-Sant Pau, Barcelona, Spain; ^3^CIBERCV, Instituto Salud Carlos III, Madrid, Spain; ^4^Second Cardiology Department, Attikon Hospital, Medical School, National and Kapodistrian University of Athens, Athens, Greece; ^5^Cardiovascular Research Chair, Autonomous University of Barcelona (UAB), Barcelona Spain; ^6^Department of Pharmacology and Pharmacotherapy, Semmelweis University, Budapest, Hungary; ^7^HCEMM-SU Cardiometabolic Immunology Research Group, Budapest, Hungary; ^8^Pharmahungary Group, Szeged, Hungary; ^9^Institute for Physiology, Justus-Liebig University Giessen, Giessen, Germany

**Keywords:** dyslipidemia, heart failure, ischaemia, LDL cholesterol, myocardial infarction, PCSK9, reperfusion

## Abstract

Extensive evidence from epidemiologic, genetic, and clinical intervention studies has indisputably shown that elevated low-density lipoprotein cholesterol (LDL-C) concentrations play a central role in the pathophysiology of atherosclerotic cardiovascular disease. Apart from LDL-C, also triglycerides independently modulate cardiovascular risk. Reduction of proprotein convertase subtilisin/kexin type 9 (PCSK9) has emerged as a therapeutic target for reducing plasma LDL-C, but it is also associated with a reduction in triglyceride levels potentially through modulation of the expression of free fatty acid transporters. Preclinical data indicate that PCSK9 is up-regulated in the ischaemic heart and decreasing PCSK9 expression impacts on infarct size, post infarct inflammation and remodeling as well as cardiac dysfunction following ischaemia/reperfusion. Clinical data support that notion in that PCSK9 inhibition is associated with reductions in the incidence of myocardial infarction, stroke, and coronary revascularization and an improvement of endothelial function in subjects with increased cardiovascular risk. The aim of the current review is to summarize the current knowledge on the importance of free fatty acid metabolism on myocardial ischaemia/reperfusion injury and to provide an update on recent evidence on the role of hyperlipidemia and PCSK9 in myocardial infarction and cardioprotection.

## Introduction

### Triglycerides (TG) and Free Fatty Acids (FA) Metabolism During Ischaemia/Reperfusion Injury (IRI)

The heart possesses a remarkably high metabolic flexibility when it comes to substrate use for adenosine triphosphate (ATP) production. At rest and during aerobic conditions, the oxidation of fatty acids (FA) highly contributes to total cardiac ATP production under physiological conditions, followed by the oxidation of carbohydrates and ketone bodies (Pascual and Coleman, 2016). In contrast, upon pathophysiological conditions with low oxygen availability, a metabolic shift toward higher oxidation of carbohydrates occurs given that glucose is the most energy-efficient myocardial substrate (Rosano et al., 2008).

Yet, if oxygen deprivation persists oxidative metabolism is repressed and anaerobic glycolysis is activated in order to spare the use of limited oxygen. The mismatch between glycolysis and glucose oxidation may result in the production of lactate and protons and the consequent fall in the pH which may impair cardiac contractility (Steenbergen et al., 1977). Besides, proton removal and sodium and calcium homeostasis lead to ATP consumption which lowers cardiac efficiency and may further deteriorate the cardiac function (Lopaschuk et al., 2010). Recently, a study in rats have also revealed that the uncoupling between glycolysis and oxidation may contribute to the development of heart failure with preserved ejection fraction (Fillmore et al., 2018). Of note, however, in the presence of free FA, lactate may improve cardiac efficiency by anaplerotic mechanisms including an enhanced tricarboxylic acid cycle combustion and reduced glycolysis rate (Lopaschuk et al., 1993).

**FIGURE 1:**
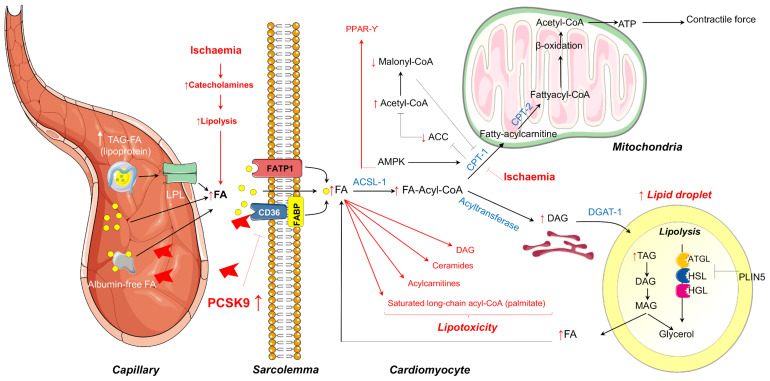
Summary of the mechanisms of myocardial FA metabolism in the healthy heart covered in this review that includes mitochondrial FA oxidation or FA storage as TG within the lipid droplets. In red are highlighted the metabolic changes that occur during ischaemia that may lead to FA overloading and consequent lipotoxicity. LPL, lipoprotein lipase; FA, fatty acid; FATP1, fatty acid transport protein 1; FABP, fatty acid binding protein; ATGL, adipose triglyceride lipase; HSL, hormone-sensitive lipase; HGL, human gastric lipase; PLIN, perilipin; CPT-1, Carnitine palmitoyltransferase-1; AMPK, AMP-dependent protein kinase; ACC, acetyl coenzyme A carboxylase; TAG, triacylglycerol; DAG, diacylglycerol; MAG, monoacylglycerol; ER, endoplasmic reticulum.

The major sources of FA are albumin-bound free FAs or FAs released from vascular lipoprotein lipase (LPL)-hydrolysis of circulating Triglycerides (TG)-rich lipoproteins (Figure 1). FAs may cross the sarcolemma by passive diffusion or by membrane-associated proteins including fatty acid transport protein (FATP), fatty acid binding protein (FABP), and fatty acid translocase (FAT/CD36), the latter being regulated in part by proprotein convertase subtilisin/kexin type 9 (PCSK9) (Van Der Vusse et al., 2000; Coort et al., 2004). Once translocated into cardiomyocytes, FAs are converted into long-chain fatty acyl-CoA which may enter the mitochondria for β-oxidation or may be stored as TG within lipid droplets which are adjacent to mitochondria (Figure 1). The heart contains significant amounts of TG within the lipid droplets in order to endogenously provide FA for oxidative metabolism for cardiac function in time of energetic needs. Lipid droplets can be catabolized by intracellular lipases [adipose triglyceride lipase (ATGL) and hormone-sensitive lipase (HSL)] and acyltransferases and, in counterpart, preserved by the action of cardiac perilipins (PLIN) 2 to 5, mainly PLIN5 (Figure 1; Kimmel and Sztalryd, 2014; Barbosa et al., 2015). Besides energy storage, TGs also serve as a transient buffer to prevent aberrant metabolism of increased FA (Evans and Hauton, 2016). Yet, when TG buffer is exceeded because of excessive FA uptake or limited FA oxidation as occurs in several pathological conditions, FA overload leads to the formation of toxic FA metabolites, such as ceramides, diacylglycerols, long-chain acyl-CoAs, and acylcarnitines, which can interfere with cellular signaling pathways leading to apoptotic cell death. Taken as a whole, alterations due to excess FA and TG accumulation are thought to contribute to cardiac dysfunction, a term referred to as cardiac lipotoxicity (D’Souza et al., 2016). Myocardial lipotoxicity has been observed in metabolic disturbances such as obesity and diabetes and its occurrence seems to precede the development of cardiac dysfunction and adverse remodeling (McGavock et al., 2007; Ernande et al., 2010). Moreover, cardiolipotoxicity has also been reported to occur in the setting of myocardial infarction and ensuing development of heart failure (Schulze, 2009). In this regard, reperfusion after a prolonged and severe ischaemic period triggers cardiac lipotoxicity which, in turn, impairs the tissue growth factor (TGF)β/TβRII/Smad2/3 signaling pathway hindering the fibrotic reparative process of the evolving scar, leading to large infarcts and cardiac dysfunction (Vilahur et al., 2012).

Multiple experimental studies in rodents and in perfusion systems have demonstrated that cardiac metabolic perturbations occur during both ischaemia and at reperfusion, and are determined by energy substrate availability as well as the regulation of substrate metabolism (Zuurbier et al., 2020). During ischaemia, the drop in oxygen supply dramatically reduces mitochondrial oxidative processes, and anaerobic glycolysis becomes an important source of ATP production in order to maintain intracellular integrity and crucial cellular functions. However, oxidation of the glycolysis end-product pyruvate is impaired during ischaemia resulting in the accumulation of lactic acid and the subsequent decline in pH and ionic disturbances (H^+^, Na^+^, and Ca^2+^) (Dennis et al., 1991). In this latter regard, the re-establishment of ion homeostasis during ischaemia entails the use of a fraction of ATP, further aggravating cardiac dysfunction. Despite the metabolic switch during ischaemia from lactate uptake to lactate production, FAs continue to be the predominant fuel for the heart. Catecholamine discharge secondary to ischaemia-related peripheral sympathetic nervous system activation promotes adipose tissue lipolysis with the ensuing increase in plasma FA concentrations and myocardial delivery (Kantor et al., 1999). In line with this phenomenon, ischaemia has been shown to induce the transcription of genes encoding for FA uptake, transport into mitochondria, and β-oxidation (Djouadi et al., 1999; Rosano et al., 2008). Moreover, during ischaemia, there is also an increase of CPT-1 activity through AMP-dependent protein kinase (AMPK)–phosphorylation (Figure 1). CPT-1 activation, besides favoring FA uptake into the mitochondria, inhibits acetyl coenzyme A carboxylase (ACC) with the consequent reduction in the conversion of acetyl-CoA to malonyl-CoA, a potent CPT-1 inhibitor. Hence, a decrease in malonyl-CoA intracellular levels allows the entry of FA into the mitochondria for oxidation, allowing ATP generation during myocardial ischaemia. A persistent reduction in oxygen supply may eventually overcome AMPK activity. As such, we have observed in a pig model of cardiac ischaemia (without reperfusion) a reduction in AMPK activation in the jeopardized myocardium after severe and complete coronary occlusion which was accompanied by enhanced detection of several markers of acute cardiac damage (Mendieta et al., 2020a,b). The temporal dynamics of AMPK activation during ischaemia in preclinical animal models deserve to be further investigated. In summary, during ischaemia, despite an increase in the rate of glycolysis, high circulating levels of FA in concurrence with higher CPT1 activity and lower malonyl-CoA content renders FAs the main source for energy requirements. Yet, while this is true for mild ischaemia, severe ischaemia has also shown to inhibit FA β-oxidation secondary to the accumulation of reducing equivalents NADH and FADH2 resulting in the accumulation of the FA metabolites stated above favoring cardiolipotoxicity (Figure 1).

During aerobic reperfusion of ischaemic myocardium, AMPK activation markedly reduces malonyl-CoA levels allowing a rapid recovery of FA β-oxidation to near pre-ischaemic values, yet, at expenses of pyruvate oxidation (Lopaschuk et al., 2010). This in concurrence with the further aggravation of ionic disturbances (mainly Na^+^ and Ca^2+^ overload) due to the rapid pH normalization upon reperfusion which primes the myocardium for further damage culminating in the impaired recovery of both cardiac function and efficiency (Jaswal et al., 2011).

### Fatty Acid Translocase CD36 and PCSK9 in IRI

There is substantial molecular, biochemical and physiologic evidence that long-chain FA transport involves a protein-mediated process. A number of FATP have been identified and some of them are co-expressed in the same tissues. Among the proteins involved in FA uptake, FAT/CD36 and FABP appear to be key transporters. Besides its sarcolemmal localization, CD36 is also stored in intracellular compartments that reversibly translocate to the plasma membrane of the cardiomyocyte in response to an increase in energy demand (Kim and Dyck, 2016). In the heart, studies in FAT/CD36 null mice have revealed that this protein is key to regulate the increase in the rate of FA metabolism (Chabowski et al., 2007) and this might be due to FAT/CD36 being a transporter itself or an indirect regulator through interaction with other free FAT in the cell membrane (Carley and Kleinfeld, 2011). In mice, CD36 is almost entirely responsible for the AMPK-mediated stimulation of long chain FA uptake into cardiomyocytes (Habets et al., 2007) and in turn, the interaction of FAs with CD36 regulates AMPK activity (Samovski et al., 2015). In humans, CD36 is needed for normal cardiac FA uptake over a range of extracellular FA concentrations from low to slightly elevated (Hames et al., 2014) and mutations of the FAT/CD36 gene with lack of CD36 protein expression reduces accumulation of long chain FAs in the human heart (Tanaka et al., 2001). Even cardiomyocyte FA uptake–delivered through exosomes–depends on CD36 (Garcia et al., 2019). Thus, there is clear evidence that CD36 is of utmost importance for free FA transport into cardiomyocytes.

In rats, sarcolemmal FAT/CD36 expression is decreased during ischaemia which is accompanied by an almost complete loss of FA oxidation, with no change in intramyocardial lipid content. Following reperfusion, the decrease in the sarcolemmal FAT/CD36 persists, but the FA oxidation rate returns to pre-ischaemic levels resulting in a decrease in myocardial triglyceride content (Heather et al., 2013). Genetic knockout of the FAT/CD36 protein reduces FFA oxidation in isolated mice hearts. On aerobic reperfusion after ischaemia, cardiac function of FAT/CD36 knockout hearts recover to the same extent as wild-type hearts (Kuang et al., 2004) or even show a reduced functional recovery (Irie et al., 2003) which can be rescued by adding medium-chain FAs which do not require CD36 for myocardial uptake. 4–6 weeks following cardiomyocyte specific ablation of CD36, hearts again have a decreased FA uptake compared to controls and a significant reduction in the intramyocardial triacylglycerol content. In contrast to the global CD36 knockout, however, hearts with a cardiomyocyte specific ablation of CD36 exhibit significantly improved functional recovery following ischaemia and reperfusion. This improved functional recovery is associated with lower calculated proton production prior to and following ischaemia compared to control hearts (Nagendran et al., 2013). A recently published review article summarizes the effect of small molecular inhibitors of enzymes in FA transport and metabolism pathways; here inhibition of FA oxidation in general reduces damage induced by ischaemia and reperfusion. The authors, however, point out that effects of FAs on cardiac ischaemia and reperfusion injury are critically dependent on the FA concentration, with detrimental effects commonly only observed at rather high levels of FAs which are usually not observed in humans during ischaemia and reperfusion episodes (for details, see Zuurbier et al., 2020).

FAs oxidation is mainly driven by the lipolysis of TG stored in the lipid droplets rather than through CD36 internalization (Kim and Dyck, 2016). Furthermore, specific cardiomyocyte deletion of CD36 has shown to improve cardiac recovery post-myocardial infarction (MI) by redirecting cardiac metabolism toward glucose utilization (Nagendran et al., 2013). Yet, whereas CD36 decline might be beneficial in the short-term post-ischaemia because of improved myocardial efficiency rates, levels of CD36 need to be restored upon glucose and intracellular TG storage depletion in order to meet cardiac energy demands (Kim and Dyck, 2016). In this regard, taking into consideration that PCSK9, which is found to be enhanced by hypoxia/reoxygenation (Yang et al., 2020), has also shown to degrade CD36 beyond the LDL-R (Demers et al., 2015), PCSK9 inhibition might hold promise as a therapeutic strategy to modulate myocardial CD36 content and expression and consequently FA metabolism levels in the setting of MI.

In this context, PCSK9 monoclonal antibodies modestly reduce plasma TG levels (Stoekenbroek et al., 2018b). Evolocumab treatment of 27,564 patients at high CVD risk modestly reduced fasting plasma TG levels by 16.2% (Sabatine et al., 2017). Same reduction levels were observed for patients with mixed hyperlipidaemia (Rosenson et al., 2016). Similarly, a *post-hoc* analysis of three Phase II clinical trials revealed equally modest reductions in plasma TG levels following alirocumab treatment in patients receiving background statin therapy (Toth et al., 2016). The effect of PCSK9 inhibitors on TG levels is contributed to the enhanced LDL-R-mediated catabolism of intermediate-density lipoproteins (IDLs) (Dijk et al., 2018).

## Effects of PCSK9 Beyond LDL Reduction: Impact on IRI

The majority of experimental studies have shown that hypercholesterolemia increases infarct size in animal models. Decreased cardiac nitric oxide content, increased oxidative/nitrosative stress, enhanced apoptotic cell death and dramatic changes in the cardiac gene expression profile, in presence of hypercholesterolemia, leads to myocardial dysfunction (Andreadou et al., 2017).

**FIGURE 2:**
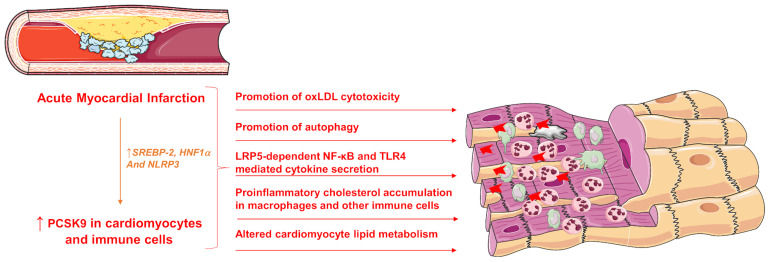
Pleiotropic effects of increased PCSK9 expression during acute MI. SREBP-2, sterol response element binding protein 2; HNF1α, hepatocyte nuclear factor 1 α; LRP5, Low-density lipoprotein receptor-related protein 5; NF-κB, Nuclear Factor kappa-light-chain-enhancer of activated B cells; TLR4, Toll-like receptor 4; PCSK9, Proprotein convertase subtilisin/kexin type 9.

PCSK9 may not only play a crucial role in binding to the LDL-R and increasing its endosomal and lysosomal degradation thus inhibiting its recycling to the cell surface but may also exhibit several, LDL-R-independent activities on a plethora of cell types, including endothelial cells, vascular smooth cells, immune cells and cardiomyocytes (Figure 2).

The role of PCSK9 regarding the function and adaptation of cardiomyocytes is under investigation. PCSK9 indirectly affects cardiomyocytes by modulating the plasma concentrations of low-density lipoprotein cholesterol (LDL-C) and oxidized LDL. Cardiomyocytes express LDL-R but it seems that low density lipoprotein receptor-related protein 1 (LRP1) and very low-density lipoprotein (VLDL) receptors are responsible for cholesterol accumulation in these cells (Cal et al., 2012). When adult rabbit ventricular cardiomyocytes are exposed to LDL-C, calcium transients through transsarcolemmal calcium transport pathways are slightly increased suggesting that LDL-C may improve cardiac function. On the other hand, in atrial-like cardiomyocytes (HL-1 cells), LDL-C decreases the expression of sarcoplasmatic reticulum (SR) calcium ATPase (SERCA) 2a, ryanodine receptor 2 (RyR2), and connexin-40 within 24 h causing a reduction in calcium transients indicating a deterioration of cardiac function (Glerup et al., 2017). The participation of PCSK9 in these effects of LDL-C was investigated in ventricular cardiomyocytes without defining the intracellular targets of PCSK9. It was reported that adult terminally differentiated ventricular cardiomyocytes constitutively express PCSK9 on the protein and mRNA level and that oxLDL-dependent effects on cell shortening can be antagonized by neutralizing PCSK9 in cardiomyocytes (Schlüter et al., 2017).

PCSK9 exerts important cardiac effects. In 2014, it was firstly reported that along with hepatic PCSK9 mRNA expression, the plasma PCSK9 concentration is drastically increased in acute MI in rats. The up-regulation of PCSK9 is driven by sterol response element binding protein 2 (SREBP-2) and hepatocyte nuclear factor 1 α (HNF1α), both of which are the predominate transcription factors for PCSK9 (Zhang et al., 2014). PCSK9 is also expressed in the myocardium and is significantly up-regulated in hypoxia/reoxygenation-stimulated primary murine cardiomyocytes compared with cells grown in normoxic environments (Ding et al., 2018; Yang et al., 2020). Again, in MI the maximum expression of PCSK9 was found in the border zone 1 week after left coronary artery (LCA) ligation (Ding et al., 2018). The fate of cardiomyocytes in the border zone of an infarction is crucial since although they experience hypoxic phases, they do not undergo cell death immediately. Thus, the enlargement of the infarct or the limitation of its extent is defined in part by the border zone, therefore the border zone cardiomyocytes appear to be promising targets for therapeutic strategies during MI (Kuhn et al., 2020). The expression of PCSK9 in cardiomyocytes was associated with the development of autophagy in the ischaemic heart (Ding et al., 2018). A utophagy is activated in cardiomyocytes early in response to hypoxia, pro-inflammatory cytokines, and angiotensin II and its attenuation can limit ischaemic injury in experimental models of myocardial ischaemia highlighting a therapeutic target of PCSK9 inhibitors. Interestingly, hearts of patients who had died of acute MI (1–7 days post-MI) also showed expression of PCSK9 and autophagy (LC3 expression) in the border zone-similar to the findings in the infarcted mouse heart (Ding et al., 2018).

Besides expression of PCSK9 in the cardiomyocytes, PCSK9 is secreted from the myocardium and is correlated to infarct size and cardiac function (Ding et al., 2018). Specifically, the sustained PCSK9 release during cardiac ischaemia and reperfusion induced serious cardiac deleterious effects including cell death and dysfunction (Ding et al., 2018). Inflammatory stimuli such as NLRP3 inflammasome-dependent activation of interleukin 1 beta (IL-1β), are considered to be powerful inducers for PCSK9 secretion in both macrophages and tissues such as heart and aorta (Ding et al., 2020). Interestingly, PCSK9 itself might be an important inflammatory mediator since PCSK9 might induce intracellular cholesterol accumulation in macrophages and several other immune cell types, increasing toll-like receptor function and amplifying certain inflammatory reactions that may lead to progression of coronary atherosclerosis (Paciullo et al., 2017; Ágg et al., 2018; Ricci et al., 2018; Kim et al., 2019; Momtazi-Borojeni et al., 2019).

The investigation on PCSK9 regulatory function in myocardial ischaemia revealed that PCSK9 activated NF-κB signaling resulting in a more pronounced secretion of pro-inflammatory cytokines such as tumor necrosis factor-α (TNF-α), interleukin-6 (IL-6) and IL-1β by macrophages worsening the hypoxia-reoxygenation-induced injury in cardiomyocytes (Yang et al., 2020). PCSK9 has been shown to promote inflammation in atherosclerosis through TLR4/NF-κB pathway and increase macrophage release of pro-inflammatory cytokines, TNF-α, IL-6, IL-1β, interferon-γ (IFN-γ), C-X-C motif ligand 2, and Monocyte Chemoattractant Protein-1 (Tang et al., 2017; Ricci et al., 2018; Macchi et al., 2019). Specifically, IFN-γ promotes and exacerbates atherosclerosis by affecting the lipid accumulation and foam cell formation in the vascular wall and altering the cellular structure in the plaque (Luo et al., 2017). Other cytokines such as IL-13 contribute to hypercholesterolemia associated with increased PCSK9 hepatic synthesis and enhanced circulating PCSK9 levels (Low et al., 2020). T he induction of PCSK9 in response to TNF-α is dependent in the suppressor of cytokine signaling 3 (SOCS3) factor. This factor has been implicated in chronic-inflammation associated with ischaemia/reperfusion injury (IRI). Transfection with small interfering RNA (siRNA) anti-signal transducer and activator of transcript 3 (STAT3), inhibited the induction of PCSK9 by TNF-α suggesting the involvement of the JAK/STAT pathway (Ruscica et al., 2016). The involvement of the inflammatory pathway of STAT3 was confirmed in a recent publication showing that upon STAT3 silencing, leptin and resistin (proinflammatory adipokines that mediate transcriptional induction of PCSK9), lose their ability to activate PCSK9 (Macchi et al., 2020). Furthermore, PCSK9 has also shown to cooperate with LRP5 in TLR4/NFkB signaling; a decreased TLR4 protein expression levels and a decreased nuclear translocation of NFκB was detected in PCSK9 silenced cells after lipid loading, indicating a down-regulation of the TLR4/NFκB pathway, showing that LRP5 facilitates macrophage lipid uptake and forms a complex with PCSK9 that up-regulates TLR4/NFκB favoring inflammation (Badimon et al., 2020). Conversely, PCSK9 siRNA lead to a reduction in inflammation by inhibiting NF-kB activation (Tang et al., 2012). Recently, it was found that PCSK9 is expressed and secreted from epicardial adipose tissue (EAT) affecting the coronary vessels and the myocardium. EAT inflammation is associated with local PCSK9 expression, regardless of circulating PCSK9 levels, suggesting that PCSK9 derived from EAT could play an additional and independent risk to CVD (Dozio et al., 2020).

However, it has to be noted that in a recent secretome analysis, PCSK6 and not PCSK9 was secreted from and expressed in primary cardiomyocytes under hypoxic conditions as well as after MI in mice and humans (Kuhn et al., 2020). PCSK6 could present a novel biomarker for cardiac fibrosis and might be a promising future therapeutic target to reduce adverse remodeling after myocardial injury.

Taken together, these data suggest that the inhibition of PCSK9 expression may suppress the inflammatory process and thereby contribute to the treatment of myocardial ischaemia.

## Role of PCSK9 Inhibition in Myocardial Infarction

### Experimental Studies on the Role of PCSK9 Inhibition in Myocardial Infarction

In animal experiments, an increased concentration of LDL-C due to dietary interventions is either associated with a reduced, unchanged or increased MI size (Andreadou et al., 2019). Explanations for a reduction in MI size by high-fat diet-induced metabolic alterations included activation of salvage kinases (Poncelas et al., 2015) or maintained low pH during initial reperfusion following ischaemia (Inserte et al., 2019), while increased MI size in hypercholesterolemia is associated with increased formation of reactive oxygen species (ROS) during reperfusion (Andreadou et al., 2017, 2019). Interestingly, even with reduced MI size functional outcome following ischaemia/reperfusion is worse in mice with hypercholesterolemia (Pluijmert et al., 2019). Additionally, hypercholesterolemia has a profound negative effect on cardioprotection of the ischaemic heart induced either by ischaemic conditioning or cardioprotective compounds (Ferdinandy et al., 2014), that might relate to the complex effects of cholesterol on myocardial redox homeostasis (Varga et al., 2013), intercellular communication (Görbe et al., 2011), autophagy (Giricz et al., 2017), and transcriptome regulation (Ágg et al., 2018).

The role of PCSK9-inhibition or -absence in MI has been studied in *in vivo* acute MI models. In an experimental study evaluating potential adverse effects of PCSK9 absence in acute MI, no significant differences in cardiac function [cardiac output, left ventricular end systolic or diastolic volume (LVESV, LVEDV), stroke volume or ejection fraction] between wild type and PCSK9 knockout (KO) mice were observed. In addition, the left ventricular wall in the infarct area was thicker in PCSK9 knockout mice, a finding that could point to a lower rate of ventricular rupture and improved outcome in patients with big infarct sizes when treated with PCSK9 inhibitors (Winter et al., 2018; Table 1). Another study showed that PCSK9 inhibition resulted in an improved cardiac function and reduced infarct size. Specifically, the PCSK9 inhibitor, Pep2-8 trifluoroacetate at a dose of 10 μg/kg was administered at different time-points in an acute MI model: before ischaemia, during ischaemia and at the onset of reperfusion. Pep2-8 trifluoroacetate exerted cardioprotective effects demonstrated by a significant reduction in infarct size and improved LV function only when given 15 min prior the onset of ischaemia. The underlying cardioprotective mechanism was attributed to the ability of the PCSK9 inhibitor to attenuate cardiac mitochondrial dysfunction and fission and decrease the apoptotic process in the ischaemic myocardium (Palee et al., 2019; Table 1). The attenuation of mitochondrial damage has also been attributed to a decreased ROS levels caused by PCSK9 inhibition. As such, recombinant mouse PCSK9 protein (mPCSK9) has shown to enhance ROS generation, whereas PCSK9 inhibition reduces ROS formation (Ding et al., 2018). Likewise, *in vitro* studies have reported that ROS stimulators enhanced PCSK9 expression whereas ROS inhibitors blocked PCSK9 expression.

**TABLE 1:** Experimental and clinical studies regarding the role of PCSK9 in myocardial infarction.

**Study**	**Model/Patients**	**Protocol/Intervention**	**Effect**
Winter et al., 2018	MI mice model: Permanent ligation of the LAD coronary artery	PCSK9 deficiency	No significant differences in cardiac function (cardiac output, left ventricular end systolic or diastolic volume (LVESV, LVEDV), stroke volume or ejection fraction), Thicker left ventricular wall in the infarct area
Palee et al., 2019	IRI rat model	Pep2−8 trifluoroacetate 10 μg/kg (a) Before ischaemia (b) During ischaemia (c) At the onset of reperfusion	Reduction in infarct size Improvement in LV function only when it was given before ischaemia
Trankle et al., 2019	Type 1 NSTEMI patients	Randomization 1:1, double-blinded study with 2 groups: (a) Alirocumab 150 mg subcutaneously within 24 h after NSTEMI (b) Placebo	Significant reduction of LDL-C levels Neutral effects on inflammatory biomarkers (follow up at 14 days)
Gencer et al., 2020	Patients with prior MI	Secondary analysis of FOURIER trial (a) Evolocumab, 140 mg every 2 weeks or 420 mg monthly (b) Placebo	Significant reduction of the risk of the composite outcome of cardiovascular death, MI, stroke, unstable angina, or coronary revascularization by 19%
Schwartz et al., 2018	ACS patients treated with statin (atorvastatin or rosuvastatin) at a high-intensity dose or at the maximum tolerated dose	Randomization, double-blinded study with 2 groups: (a) Alirocumab 75 mg subcutaneously (b) Placebo	Significant reduction of the risk of a composite of death from coronary heart disease, non-fatal MI, fatal or non-fatal ischemic stroke, or unstable angina requiring hospitalization
Wiviott et al., 2020	Patients with stable atherosclerotic disease receiving statin therapy	Pre-specified analysis of the FOURIER trial (a) Evolocumab, 140 mg every 2 weeks or 420 mg monthly (b) Placebo	Reduction of Type 1 and Type 4 MIs
White et al., 2019	Patients with recent ACS and elevated LDL-C	Pre-specified analysis of the ODYSSEY OUTCOMES trial (a) Alirocumab 75 mg every 2 weeks (b) Placebo	Reduction of Type 1 and Type 2 MIs

*ACS, Acute Coronary Syndrome; FOURIER, Further Cardiovascular Outcomes Research With PCSK9 Inhibition in Subjects With Elevated Risk, IRI, Ischaemia/Reperfusion Injury (IRI); LAD, Left Anterior Descending; LDL-C, Low-density lipoprotein cholesterol; MI, Myocardial Infarction; NSTEMI, non-ST elevation MI.*

In summary, although very few studies have investigated the role of PCSK9 on myocardial infarct size in experimental animal models, it seems that the pharmacological inhibition of PCSK9 results in cardioprotective effects.

### Clinical Studies on the Role of PCSK9 Inhibition in Myocardial Infarction

Patients suffering from a MI are considered at very high risk for subsequent cardiovascular (CV) events since the reported 5-year risk of recurrent MI or fatal coronary heart disease (CHD) for those aged >65 years is 22% (Go et al., 2014). Due to the high burden of recurrent CV events, clinicians have to investigate new strategies for risk reduction in these patients. A further, rapid LDL-C reduction within 2–4 weeks following an acute MI beyond current standard care, including high-dose statins, seems an appealing option in order to reduce CV risk. Although statins decrease LDL-C levels by 1 mmol/L which is translated to an approximate 20% reduction in the rate of MI, the most potent hypolipidemic agents, PCSK9 inhibitors evolocumab and alirocumab that reduce LDL-C by >1 mmol/L below statin-treated levels, could further reduce the risk of MI (Trankle and Abbate, 2016). Low LDL-C levels are directly correlated with reduced risk of atherosclerotic cardiovascular disease. The pharmacological inhibition of PCSK9 has led to indisputable benefits in terms of LDL-C and CV-risk lowering. Interestingly, measuring PCSK9 circulating levels has become important. Many studies support that pharmacological therapy, i.e., statins modulate PCSK9 gene expression and protein levels providing a pointer to estimate possible alterations in the overall response to lipid-lowering medications in patients at high CV risk (Macchi et al., 2019). Thus, in August 2019, a joint consensus statement from the European Society of Cardiology (ESC) and European Atherosclerosis Society (EAS) recommended a stricter LDL-C level target for patients with recent MI [below the 1.4 mmol/L (<55 mg/dL)]. In a recent study with 25,466 patients who had attended a follow-up visit 6–10 weeks after an MI event, it was shown that half of this cohort were eligible to PCSK9 inhibitors according to updated ESC/EAS guidelines (Allahyari et al., 2020). With regard to safety, very low LDL-C concentrations do not significantly increase risk of adverse events (e.g., new-onset diabetes mellitus, elevations in hepatic transaminases, muscle symptoms and increase in neurocognitive events) (Giugliano et al., 2017). Whether PCSK9 inhibition has beneficial effect specifically on patients with MI has to be established in clinical trials. Currently, we have limited data in this regard. Results from clinical studies have already shown that PCSK9 inhibitors have different effects on type and size of myocardial infarcts. For example, evolocumab reduced the risk of spontaneous and procedural MI, but had no effect on type 2 (myocardial oxygen supply demand mismatch) MI events. The benefit for evolocumab was consistent for myocardial infarcts generally considered more severe, including ST-elevation myocardial infarction (STEMI) and those with larger elevations of cardiac biomarkers (Wiviott et al., 2020). On the other hand, alirocumab added to intensive statin therapy was found to attenuate the risk of type 2 myocardial infarct events (White et al., 2019).

Alirocumab may benefit patients who have had an MI and are at very high risk of recurrent CV events since LDL-C target levels can be achieved, regardless of MI or ischemic stroke status (Bruckert et al., 2019). Also, in these high-risk patients that were included in the study, alirocumab was well tolerated with a favorable safety profile [similar treatment-emergent adverse events (TEAEs) were observed among groups], regardless of previous history of MI/ischaemic stroke (Bruckert et al., 2019).

In a pilot study, it was shown that alirocumab administration at the time of non-ST elevation MI (NSTEMI) significantly reduced LDL-C levels at 14 days but had neutral effects on inflammatory biomarkers such as high-sensitivity C-reactive protein (hsCRP), IL-6, and TNF-a (Trankle et al., 2019; Table 1).

An important question that should be addressed is whether the PCSK9 inhibition has direct effect on CV outcomes in patients with MI. In a secondary analysis of the FOURIER trial including 5,711 patients with a recent MI, evolocumab significantly reduced the risk of the composite outcome of cardiovascular death, MI, stroke, unstable angina, or coronary revascularization by 19% (Gencer et al., 2020; Table 1). The results for MI patients would be more impressive if evolocumab would be found effective in reducing the area and severity of MIs. It is known that larger MIs are associated with increased risk, suggesting that a robust reduction of larger MIs would lead over time to a mortality reduction (Wiviott et al., 2020). Such an effect in mortality has been confirmed over a longer duration of follow-up (2.8 years) in a study that involved 18,924 ACS patients treated with alirocumab subcutaneously at a dose of 75 mg (Schwartz et al., 2018; Table 1).

In a recent analysis of FOURIER trial, evolocumab reduced the number of atherothrombotic (Type 1) MI and consistent reductions were seen for type 1 and PCI-related (Type 4) MIs, STEMI, and NSTEMI, and those with higher troponin levels (Wiviott et al., 2020; Table 1). In a pre-specified analysis from the ODYSSEY OUTCOMES trial, alirocumab added to intensive statin therapy during 2.8 years of follow-up reduced the occurrence of both Type 1 and due to myocardial supply and/or demand imbalance (Type 2) MIs in patients with recent (1–12 months) ACS and elevated LDL-C. However, there was no apparent effect of alirocumab on reducing Type 4 MIs which is mainly attributed to differences in patient populations’ enrollment (high-risk patients versus recent ACS patients) and duration of follow-up (2.8 versus 2.2 years in the FOURIER trial) (White et al., 2019; Table 1).

In a recent meta-analysis of 67 randomized controlled trials (RCTs) including 259,429 participants PCSK9 inhibitors plus statin significantly reduced the risk of non-fatal MI (RR 0.82, 95% CI 0.72–0.93, *p* = 0.003) or stroke (RR 0.74, 95% CI 0.65–0.85, *p* < 0.001) (Chaiyasothi et al., 2019).

Inclisiran, a long acting synthetic siRNA that targets hepatic PCSK9 synthesis, produces similar reductions in LDL-C as PCSK9 monoclonal antibodies (Board et al., 2020; Landmesser et al., 2020). In a recent meta-analysis of three RCTs comparing cardiovascular outcomes in patients with hypercholesterolemia treated with maximally tolerated dose statins with or without additional lipid-lowering therapy (*n* = 3,783 patients), it was found that there was no statistically significant difference in the risk of MI in patients randomized to inclisiran compared with placebo (Asbeutah et al., 2020). However, ORION-4 trial has been designed as a “cardiovascular outcomes trial” and will assess the effects of inclisiran on clinical outcomes in approximately 15,000 subjects with atherosclerotic cardiovascular disease. The results of this study will shed light to the effect of inclisiran on the MI (Stoekenbroek et al., 2018a). Increased Lipoprotein (Lp) (a) levels are prothrombotic and associated with MI. PCSK9 inhibitors are the only drugs reducing increased Lp(a) levels by 25 to 30% (Shapiro et al., 2019). Kinetic studies have revealed the mechanism of action of evolocumab and alirocumab lower plasma Lp(a) concentration. Evolocumab decreases the production (as monotherapy) and increases clearance (in combination with atorvastatin) of Lp(a) particles. Increased clearance of Lp(a) with PCSK9 inhibition may relate to supraphysiological up-regulation of hepatic LDL-Rs and/or decreased competition of Lp(a) with LDL particles for LDL-R uptake (Watts et al., 2018). Alirocumab efficiently reduces circulating apoB100 levels primarily by enhancing its catabolism and apo(a) levels primarily by lowering its production (Croyal et al., 2018). Future studies should examine if these new therapies that lower Lp(a) could reduce the risk for future MI. The reduction of Lp(a) by PCSK9 inhibitors is not always in parallel with the respective reduction of LDL-C, a finding suggesting a direct effect of PCSK9 on reducing Lp(a) production and not necessarily through an effective reduction of LDL-C levels (Shapiro et al., 2019).

In conclusion, the provided data suggests that PCSK9 inhibition has a beneficial role in patients with MI. The mechanism by which this occurs is under investigation. It has been postulated that better outcomes may derive from a rapid LDL-C reduction (stabilizing the LDL-R), a diminishment of the pro-inflammatory signaling by potentially reducing those LDL particles susceptible to oxidation or through Lp(a) decline, and/or enhanced removal of the pro-inflammatory apoptotic debris.

## Conclusion

Hypercholesterolemia has profound negative effect on cardioprotection and PCSK9 has emerged as a therapeutic target for reducing plasma LDL-C. Taking into consideration that MIs of increasing size are associated with a significant increase in the risk of death, a detailed description of the effect of PCSK9 in infarct size and cardiac function is very important. The results of clinical trials indicate that PCSK9 inhibitors are beneficial across multiple MI subtypes.

However, the effect of PCSK9 inhibitors on cardioprotective cellular mechanisms needs to be tested to reveal if these group of drugs may promote cardioprotection (Ferdinandy et al., 2014) or possess some hidden cardiotoxic effect (Ferdinandy et al., 2019). Experimental studies would help to shed light on these open questions. Future studies should be conducted in order to examine different agents, doses, time of administration for more conclusive results regarding the role on PCSK9 inhibitors in cardiac IRI and cardioprotection from it.

## Author Contributions

All authors contributed to the content of the manuscript, have read and approved the final version of the manuscript. IA and RS finally revised the manuscript.

## Conflict of Interest

PF is the founder and CEO of Pharmahungary Group, a group of R&D companies. LB declares to have acted as SAB member of Sanofi, Bayer, and AstraZeneca, has a Research Grant of AstraZeneca, speaker fees of Lilly, MSD-Boerhinger and AstraZeneca and to have founded the Spin-off Glycardial Diagnostics SL. GV has a Research Grant of AstraZeneca and is founder of the Spin-off Glycardial Diagnostics SL. IA, II, and MT declare that they have not a potential conflict of interest. The remaining authors declare that the research was conducted in the absence of any commercial or financial relationships that could be construed as a potential conflict of interest.
